# Merkmale der Inanspruchnahmepopulation einer Eltern-Kind-Station: Frühe interaktionszentrierte Behandlung als Chance in der Kinder- und Jugendpsychiatrie

**DOI:** 10.1007/s40211-022-00430-3

**Published:** 2022-09-21

**Authors:** Christina Taferner, Ann-Christin Jahnke-Majorkovits, Sanae Nakamura, Kathrin Sevecke

**Affiliations:** 1 Kinder- und Jugendpsychiatrie, Psychotherapie und Psychosomatik, A.ö. Landeskrankenhaus Hall, Milser Straße 10, Haus 6, 6060 Hall in Tirol, Österreich; 2 Medizinische Universität Innsbruck, Anichstr. 35, 6020 Innsbruck, Österreich

**Keywords:** Eltern-Kind, Station, Populationsmerkmale, DC:0–5, Child Behavior Checklist, Elternbelastungsinventar, Parent-child unit, Population characteristics, DC:0–5, Child Behavior Checklist, Parent Stress Inventory

## Abstract

**Grundlagen:**

Bei einer stationären kinderpsychiatrischen Eltern-Kind Behandlung wird das Kind als Patient und die Eltern als Begleitperson aufgenommen, um der Bedeutung der Eltern-Kind-Interaktion bei der Entstehung und Aufrechterhaltung von psychischen Störungen von Kindern gerecht zu werden und Eltern in die Behandlung von Kindern mit emotionalen und/oder Verhaltensauffälligkeiten zentraler zu integrieren. Um diese Behandlungsform zukünftig weiter auszubauen und effektiver zu gestalten, wurden in der vorliegenden Studie die Merkmale der bisherigen Inanspruchnahmepopulation der kinderpsychiatrischen Eltern-Kind-Station in Tirol hinsichtlich der kindlichen Symptomatik mittels ICD-10 und DC:0–5 und der Belastung der Eltern genauer untersucht.

**Methodik:**

Zur Überprüfung der kindlichen Symptomausprägung sowie der Belastung der Eltern wurden die Ergebnisse der Child Behavior Checklist 1 ½–5, der Child Behavior Checklist 6–18R sowie des Eltern-Belastungsinventars von Tröster (2011) von 96 Eltern-Kind-Paaren herangezogen.

**Ergebnisse:**

Die 6–10-jährigen Patienten wiesen bei der Gesamtskala des CBCL höhere T‑Werte (M = 76,9, SD = 7,1) auf als die 0–5-jährigen Patienten (M = 63,1, SD = 12,4), t (50) = −3,52, *p* < 0,001. Bei der EBI-Gesamtskala unterschieden sich die 0–5-jährigen Patienten und die 6–10-jährigen Patienten hinsichtlich der T‑Werte nicht, t (54) = −0,75, *p* = 0,459, ebenso wie im EBI-Kinderbereich t (54) = −1,75, *p* = 0,087 und im EBI-Elternbereich, t (54) = 0,19, *p* = 0,846. Auch die vier Diagnosegruppen unterschieden sich weder in der Gesamtskala des EBI, F (4,58) = 1,34, *p* = 0,266, noch im Elternbereich des EBI, F (4,58) = 1,44, *p* = 0,232, oder im Kinderbereich des EBI, F (4,58) = 2,81, *p* = 0,033.

**Schlussfolgerungen:**

Eine frühzeitige Erkennung und Behandlung von Verhaltensauffälligkeiten bzw. psychischen Störungen bei sehr jungen Kindern scheint maßgeblich, um langfristige negative Folgen sowie eine Chronifizierung zu verhindern. Eltern-Kind-Therapien sollten im Allgemeinen auf die Aufdeckung und Veränderung aktueller dysfunktionaler Interaktionsmuster zwischen Eltern und Kind fokussieren.

## Abstract (en)

**Background:**

In inpatient child psychiatric parent-child treatment, the child is admitted as a patient and the parents as accompanying persons. Due to the importance of parent-child interaction in the development and maintenance of mental disorders in children, parents are integrated more centrally in the treatment of children with emotional or behavioral disorders. In order to further expand this form of treatment in the future and to make it more effective, the characteristics of the previous utilization population of a child psychiatric parent-child unit in Tyrol were examined with regard to child symptomatology and the burden on parents.

**Methods:**

The Child Behavior Checklist 1 ½–5, Child Behavior Checklist 6–18R, and Tröster’s (2011) Parent Stress Inventory scores of 96 parent-child pairs were used to examine child symptom expression and parent stress.

**Results:**

The 6–10 year old patients had higher T‑scores (M = 76.9, SD = 7.1) than the 0–5 year old patients (M = 63.1, SD = 12.4) on the CBCL total scale, t (50) = −3.52, *p* < 0.001. On the EBI total scale, the 0–5 year old patients and the 6–10 year old patients did not differ in terms of T‑scores, t (54) = −0.75, *p* = 0.459, as well as in the EBI child domain t (54) = −1.75, *p* = 0.087 and in the EBI parent domain, t (54) = 0.19, *p* = 0.846. Also, the four diagnostic groups did not differ in the EBI total scale, F (4,58) = 1.34, *p* = 0.266, nor in the parent domain of the EBI, F (4,58) = 1.44, *p* = 0.232, nor in the child domain of the EBI, F (4,58) = 2.81, *p* = 0.033.

**Conclusions:**

Early identification and treatment of behavioral or mental disorders in very young children seems crucial to prevent long-term negative consequences as well as chronicity. Parent-child therapies should generally focus on identifying and changing current dysfunctional patterns of interaction between parent and child.

## Hintergrund

Psychische Störungen bei jungen Kindern unterscheiden sich von denen bei älteren Kindern durch eine entwicklungsspezifische Symptomatik und durch eine hohe Bedeutung der Interaktion mit Bezugsperson [1]. Im Vorschulalter wird der Eltern-Kind-Beziehung bzw. der Eltern-Kind-Interaktion sowohl bei der Entstehung als auch der Aufrechterhaltung der psychischen Symptomatik des Kindes eine zentrale Bedeutung beigemessen. Problematische Interaktions- und Beziehungsmuster können zu anhaltenden verhaltensregulatorischen Problemen eines Säuglings und Kleinkindes [2] und einer eingeschränkten Responsivität bzw. Feinfühligkeit der Eltern [3] führen. Dies wirkt sich wiederum negativ auf die Beziehungsgestaltung zwischen Eltern und Kindern aus [4, 5]. Vor diesem Hintergrund wurden neben vielzähligen ambulanten interaktionszentrierten Behandlungsprogrammen und der gemeinsamen (erwachsenenpsychiatrischen) stationären Behandlung von psychisch kranken Müttern und ihren Säuglingen auch Behandlungskonzepte zu kinderpsychiatrischen Eltern-Kind-Stationen entworfen. Bei diesen Stationen wird das Kind als Patient und die Eltern als Begleitperson aufgenommen. So konnte beispielsweise in einer Studie mit 60 Familien aufgezeigt werden, dass sich am Ende einer kinderpsychiatrischen stationären Eltern-Kind-Behandlung sowohl die Symptome der Kinder als auch das Stresserleben der Eltern signifikant reduzierten [6]. Müller et al. konnten durch eine familientagesklinische Behandlung eine Symptomverbesserung bei Kleinkindern [7] und eine reduzierte mütterliche Symptomatik nach der gemeinsamen Behandlung [8] aufzeigen. Eine Abnahme des Belastungserlebens sowie die Veränderungen des Erziehungsverhaltens nach einer gemeinsamen vierwöchigen Behandlung blieben auch bei einer Follow-Up Messung nach vier Wochen stabil [9]. Gleichzeitig stehen die psychischen Auffälligkeiten der Kinder aber auch mit einer erhöhten Belastung der Eltern im Zusammenhang, die im Sinne einer bidirektionalen Interaktion zu einer wechsel- oder einseitigen Überforderung führen kann [10]. Somit können sich Belastungen bei den Eltern, bei den Kindern oder zwischen Eltern und Kind manifestieren. Eine erhöhte elterliche Stressbelastung ist assoziiert mit elterlichen psychischen Erkrankungen und ungünstigen Erziehungspraktiken. Besteht bei einem Elternteil eine psychische Erkrankung ist das Risiko des Kindes, ebenfalls psychisch zu erkranken, deutlich erhöht [11]. Vor diesem Hintergrund sollten die (psycho-)therapeutischen Interventionen im Säuglings- und Kleinkindaltert nicht nur die hohe Entwicklungsdynamik, sondern auch die Bedeutung der Interaktion zwischen dem Kleinkind und seiner sozialen Umwelt maßgeblich berücksichtigen. Die elterlichen Bedürfnisse und Ressourcen sind dabei von zentraler Bedeutung. In Hinblick auf kindsbezogene Aspekte zeigen die Ergebnisse der 2. Erhebungswelle der KiGGS-Studie des Kinder- und Jugendgesundheitssurveys des Robert-Koch-Instituts erhöhte Prävalenzen für psychische Erkrankungen bei älteren Kindern im Alter von 9 bis 11 Jahren im Vergleich zu den 3–5-jährigen. Zudem sind Jungen häufiger von psychischen Störungen betroffen als Mädchen [12]. Global zeigt sich jedoch, dass allgemeine Prävalenzzahlen für die frühe Kindheit relativ einheitlich zwischen 12,5 und 18 % liegen. Das bedeutet, dass psychische Störungen im Vorschulalter genauso häufig auftreten, wie bei älteren Kindern und Jugendlichen [13]. Durch die Erfassung der kinds- und elternbezogenen Merkmale der bisherigen Inanspruchnahmepopulation der Eltern-Kind-Station in Tirol soll der Bedarf der Eltern-Kind-Behandlung verdeutlicht werden, zukünftige Behandlungsstrategien abgeleitet sowie die bereits etablierten Strukturen verbessert werden.

### Rahmenbedingungen der kinderpsychiatrischen Eltern-Kind-Station in Tirol

Bei der kinderpsychiatrischen Eltern-Kind-Station unserer Klinik wird das Kind mit psychischen Auffälligkeiten mit einem Elternteil als Begleitperson innerhalb der Abteilung für Kinder- und Jugendpsychiatrie, Psychotherapie und Psychosomatik aufgenommen. Die durchschnittliche Aufenthaltsdauer beträgt 3 bis 4 Wochen. Durch das multimodale Behandlungsangebot mit einem Fokus auf eltern-, kinds- und beziehungsorientierten Elementen [14, 15] soll die Qualität der Eltern-Kind-Beziehung verbessert und die kindliche Symptomatik reduziert werden. Nach der Aufnahme erfolgt neben der videobasierten Diagnostik [16] der Eltern-Kind-Interaktion in Spiel-Trennungs- und Essenssituationen bei Bedarf eine ausführliche (Entwicklungs‑) Diagnostik des Kindes (Anamnese, Fragebögen. Erstellung des psychopathologischen Befunds, körperliche Untersuchung). In der Diagnosestellung wird mit dem multiaxial aufgebauten DC:0–5 (Diagnostische Klassifikation seelischer Gesundheit und Entwicklungsstörungen der frühen Kindheit) [17] als Ergänzung zu der ICD-10 [18] gearbeitet. Die DC:0–5 ist ein speziell für 0–5-Jährige entwickeltes Klassifikationssystem, welches fünf Achsen zur Erfassung der komplexen Entwicklungsdynamik des Säuglings, Kleinkind- und Vorschulalters vorsieht. Auf *Achse I* werden klinischen Störungen erfasst. Die *Achse II* ist auf den Beziehungskontext und die *Achse III* auf die medizinischen Diagnosen ausgerichtet. Auf *Achse IV* werden die psychosozialen Stressoren erfasst und mittels *Achse V* werden die Entwicklungskompetenzen der Kinder dokumentiert. Im Unterschied zur ICD-10 und zum DSM‑5 berücksichtigt die DC:0–5 bei der Diagnosestellung altersspezifische Besonderheiten, entwicklungspsychologische Zusammenhänge, schnelle Veränderungen in der sozioemotionalen Entwicklung und fokussiert auf die Bedeutung des Beziehungskontextes für die frühe Kindheit [19].

Darüber hinaus werden spezifische therapeutische Behandlungen für die Kinder (Spiel‑/Psychotherapie, Ergo‑, Logo- und Physiotherapie), Eltern- und Familiengespräche, sowie Gruppenbehandlungen angeboten. Zu den therapeutischen Gruppenangeboten zählt die Eltern-Kind-Interaktionsgruppe, basierend auf dem bindungsorientierten Konzept *Watch-Wait–Wonder *[20]. Ebenso wird eine integrative klinische Tanztherapie für Eltern und Kindern gemeinsam im Gruppensetting angeboten. Für Kinder mit Problemen im Essverhalten kann in der *Pic-Nic-Gruppe* ein spielerischer und sensorischer Zugang zu Nahrungsmitteln und der Esssituation aufgebaut werden. Die Elterngruppe bietet einen Rahmen, Bedürfnisse und Befindlichkeiten der Eltern zu thematisieren, psychoedukative Konzepte einzubauen sowie einen Erfahrungsaustausch zu ermöglichen. Bei Einzelgesprächen mit den Eltern werden biografische Erfahrungen sowie das emotionale Erleben in der Elternrolle im Hinblick auf den Einfluss auf die elterliche Feinfühligkeit und Aufrechterhaltung der kindlichen Symptomatik thematisiert und reflektiert. Des Weiteren findet wöchentlich eine Entspannungsgruppe für die Eltern statt. Aus dem Pflege- und sozialpädagogischem Bereich wird eine Kinderbetreuungsgruppe, sowie die Alltags- und Ausflugsgruppe angeboten. Die Kochgruppe stellt einen weiteren Baustein des Gruppenangebots dar, die gemeinsam mit Müttern/Vätern und ihren Kindern stattfindet.

## Methoden

### Vorgehen

**Figure 1:**
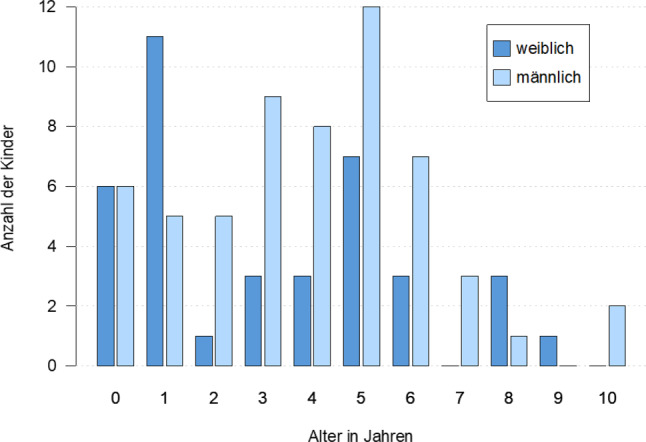

Die Eltern aller Patienten unterzeichneten vor der Teilnahme an der Untersuchung eine Einwilligungserklärung nach einer ausführlichen Patienteninformation. Die Studie wurde von der zuständigen Ethikkommission begutachtet und mit einem positiven Votum versehen (EK Nr: 1432/2020). Es handelte sich dabei um 96 Eltern-Kind-Paare mit einem mittleren Alter von 3,6 Jahren (Altersspanne von 3 Monaten bis 10 Jahre; siehe Abb. 1). Die Diagnostik, die von den Stationspsychologinnen durchgeführt wurde, erfolgte nach den aktuellen AWMF-Leitlinien [27] und umfasste bei allen Kindern Anamnese, Fragebögen, psychopathologischer Befund, Entwicklungsdiagnostik und körperliche Untersuchung sowie additiv eine Video-Interaktions-Diagnostik. Für die vorliegende Arbeit wurden die Ergebnisse der CBCL und des EBI zur Untersuchung der Symptomausprägung der Kinder und der elterlichen Belastung herangezogen. Die Diagnosen wurden nach klinischen Kriterien nach ICD-10 und zusätzlich (ab der deutschsprachigen Erscheinung 2019) nach DC:0–5 vergeben. Die Achsen‑1 der jeweiligen Diagnosemanuale wurden detailliert ausgewertet. Es wurden nur gesicherte Diagnosen, keine Verdachtsdiagnosen berücksichtigt.

### Beschreibung der Stichprobe

Es wurde die gesamte Inanspruchnahme-Population aller neu vorgestellten PatientInnen der Eltern-Kind-Station an der Abteilung für Kinder- und Jugendpsychiatrie, Psychosomatik und Psychotherapie der Universitätsklinik Innsbruck/Hall, im Zeitraum von Jänner 2018 bis Dezember 2020, eingeschlossen. Aus dem ursprünglichen Datensatz mit 97 Kindern wurde ein 13-jähriges Geschwisterkind entfernt, da dieses Alter mehr als zwei Standardabweichungen vom Alter der Zielgruppe (0–5 Jahre) der Eltern-Kind-Station entfernt ist. Die verbliebenen *N* = 96 Kinder im Datensatz wurden nach Altersgruppe (Zielgruppe: 0–5 Jahre, erweiterte Gruppe: 6–10 Jahre) und dem Aufnahmegrund (Patient oder begleitendes Geschwisterkind) in eine von vier Subgruppen (0–5-jährige Patienten, 6–10-jährige Patienten, 0–5-jährige Geschwisterkinder, 6–10-jährige Geschwisterkinder) klassifiziert. In den statistischen Analysen wurden nur die klinisch auffälligen Patienten miteinbezogen. Die Geschwisterkinder scheinen in Grafiken und der deskriptiven Gesamtdarstellung der Patientenpopulation auf, um ein realistisches und differenziertes Bild der Familien auf Station abbilden zu können. Unter den insgesamt 96 Kindern, die jeweils mit einer Bezugsperson aufgenommen wurden, befanden sich 38 weibliche Patientinnen und 58 männliche. Das Durchschnittsalter der aufgenommenen PatientInnen betrug *M* = 3,6 Jahre (*SD* = 2,5). Die Altersverteilung nach Geschlecht ist in Abb. 1 zu sehen.

**Figure 2:**
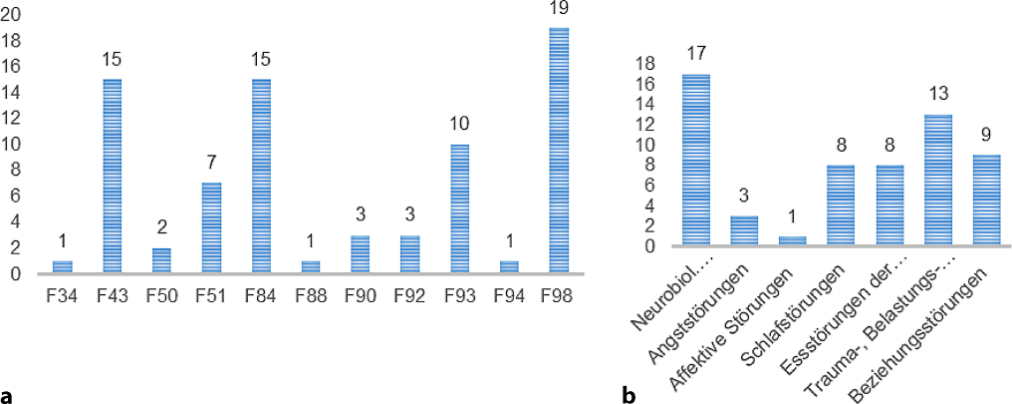

87 % (*n* = 83) der Kinder wurden erstmalig aufgenommen, bei 12 % (*n* = 11) handelte es sich bereits um die zweite Aufnahme. Die durchschnittliche Aufenthaltsdauer des Erstaufenthaltes betrug 28 Tage. 74 % (*n* = 71) der Kinder wurden während des stationären Aufenthaltes von ihren Müttern begleitet, 7 % (*n* = 7) von ihren Vätern und 4 % (*n* = 4) von ihrer Pflegemutter, bei 15 % (*n* = 14) nahmen beide Elternteile (abwechselnd) an der stationären Behandlung teil. Die meisten Kinder (71 %, *n* = 65) hatten Geschwister, die in 39 % (*n* = 37) der Fälle auch mitaufgenommen wurden und die ebenfalls mittels Diagnosemanual diagnostiziert und multimodal behandelt wurden. Die Zuweisung zur Eltern-Kind-Station erfolgte am häufigsten durch die Ambulanz der KJP (37 %, *n* = 35), gefolgt von der Ambulanz der psychologischen Säuglings- und Kleinkindberatung (23 %, *n* = 22), der Kinder- und Jugendhilfe (10 %, *n* = 10) sowie sonstigen Zuweisungen (andere Landeskrankenhäuser, Fachärzte, Eigeninitiative) in 30 % der Fälle (*n* = 29). Die Diagnosen werden nach ICD-10 (Abb. 2a) und DC:0–5 (Abb. 2b) dargestellt.

### Erhebungsinstrumente

Die Child Behavior Checklist 1 ½–5 (CBCL 1 ½–5) von Achenbach und Rescorla [21] ist ein, für den Altersbereich von 18 Monaten bis fünf Jahre, standardisiertes und normiertes Fragenbogenverfahren zum Symptomscreening mit 99 Problem-Items. Die Child Behavior Checklist 6–18R von Achenbach [22] stellt ein Fremdbeurteilungsverfahren durch die Eltern für Kinder und Jugendliche von 6–18 Jahren dar, welches der Erfassung von Verhaltensauffälligkeiten, emotionalen Auffälligkeiten, somatischen Beschwerden sowie sozialen Kompetenzen von Kindern und Jugendlichen im Schulalter aus Sicht der Eltern dient. Die Einschätzung des kindlichen Verhaltens erfolgt bei diesen Fremdbeurteilungsbögen für Eltern oder ErzieherInnen auf einer dreistufigen Skala (von 0 = „nicht zutreffend (soweit bekannt)“, 1 = „etwas oder manchmal zutreffend“, und 2 = „genau oder häufig zutreffend“). Die Items sind beim CBCL 1 ½–5 sieben Problemskalen (Emotionale Reaktivität, Ängstlich/Depressiv, Körperliche Beschwerden, Sozialer Rückzug, Schlafprobleme, Aufmerksamkeitsprobleme und Aggressives Verhalten) und drei übergeordneten Skalen (Externale Probleme, Internale Probleme und Gesamt) zuordnen. Die interne Konsistenz bei der vorliegenden Stichprobe betrug Cronbachs $\upalpha =0{,}95$ für externale Probleme und $\upalpha =0{,}84$ für internale Probleme. Bei der Auswertung der CBCL 6–18R werden acht Problemskalen gebildet (Ängstlich/depressiv, Rückzüglich/depressiv, Körperliche Beschwerden, Soziale Probleme, Denk-, (Schlaf-) und repetitive Probleme, Aufmerksamkeitsprobleme, Regelverletzendes Verhalten und Aggressives Verhalten) sowie drei übergeordnete Skalen berechnet (Gesamt, Internale Probleme, Externale Probleme). Die interne Konsistenz bei der vorliegenden Stichprobe betrug Cronbachs $\upalpha =0{,}88$ für externale Probleme und $\upalpha =0{,}92$ für internale Probleme.

Das Eltern-Belastungsinventar (EBI) von Tröster [23] stellt die deutsche Version des Parenting Stress Index (PSI) dar. In der vorliegenden Untersuchung wurde das EBI verwendet, um das elterliche Belastungsausmaß zu erfassen und die Elternbelastung hinsichtlich der unterschiedlichen Störungsbilder zu vergleichen. Elterliche Belastung wird in diesem Fragebogen als Stress der Eltern definiert, wenn ein subjektives Missverhältnis aus benötigten und vorhandenen Ressourcen der Kindererziehung vorliegt [10, 24]. Vor dem Hintergrund der Annahme, dass die elterliche Belastung zum einen aus schwierigen Verhaltensweisen des Kindes (kindlichen Belastungsquellen) und zum anderen aus eigenen Beeinträchtigungen (elterliche Belastungsquellen) resultieren kann, enthält der EBI Items zu den 5 Primärskalen Anpassungsfähigkeit, Stimmung, Anforderung, Akzeptierbarkeit und Ablenkbarkeit/Hyperaktivität des Kindes (= Kindbereich) sowie Items zu den sieben Primärskalen Partnerbeziehung, Depression, Persönliche Einschränkung, Bindung, Zweifel an der elterlichen Kompetenz, soziale Isolation, Gesundheit der Eltern (= Elternbereich). Für die Sekundärskalen (Eltern- und Kinderbereich) sowie den EBI-Gesamtwert liegen T‑Wert Normen vor. Aus dem Belastungsprofil können anschließend Interventionsvorschläge abgeleitet werden. Der EBI besteht aus 48 Items mit Likert-Skala (trifft gar nicht zu = 1 bis trifft genau zu = 5). Die interne Konsistenz bei der vorliegenden Stichprobe betrug Cronbachs $\upalpha =0{,}95$ für die Gesamtskala, $\upalpha =0{,}91$ für den Kinderbereich und $\upalpha =0{,}93$ für den Elternbereich.

### Statistische Analysen

Die statistische Analyse erfolgte mit IBM SPSS Statistics Version 26 und R Version 4.1.1. Deskriptive Statistiken wurden für die soziodemografischen Merkmale und für die Skalen zur psychischen Gesundheit berechnet.

Die CBCL T‑Werte für die Gesamtskala, die internalen Probleme und die externalen Probleme wurden in klinisch unauffällig (≤ 59), klinischer Grenzbereich (60–63) und klinisch auffällig (≥ 64) klassifiziert. Die CBCL T‑Werte für die Subskalen wurden in klinisch unauffällig (≤ 64), klinischer Grenzbereich (65–69) und klinisch auffällig (≥ 70) klassifiziert.

Für die EBI T‑Werte der Gesamtskala, des Kinderbereichs und des Elternbereichs liegen keine Normwerte vor. Die EBI T‑Werte wurden deshalb entsprechend einer klinischen Diagnostik in durchschnittlich (≤ 59), überdurchschnittlich (≤ 69) und klinisch (≥ 70) klassifiziert.

Bei der induktiven Statistik wurden t‑Tests für den Vergleich zweier Gruppen und einfaktorielle Varianzanalysen mit anschließenden post-hoc-t-Tests beim gleichzeitigen Vergleich mehrerer Gruppen berechnet. P‑Werte ≤ 0,01 werden als statistisch signifikant betrachtet (zweiseitige Nullhypothesentests).

## Ergebnisse

### Symptomausprägung der Kinder

**Figure 3:**
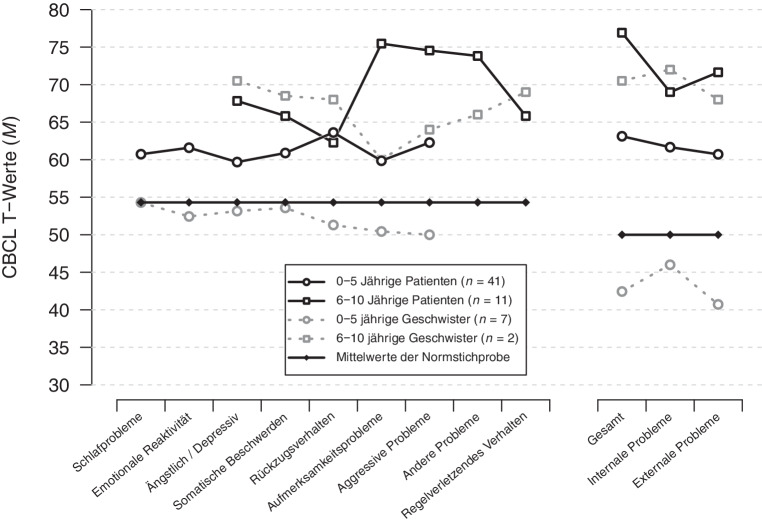

Abb. 3 zeigt die Ausprägung der einzelnen CBCL-Skalen sowie der Hauptskalen (internalisierende vs. externalisierende Verhaltensweisen) für die vier Subgruppen.

Betrachtet man die Gesamtskala des CBCL sind in der Subgruppe der 0–5-jährigen Patienten 18 (44 %) unauffällig, 12 (29 %) im klinischen Grenzbereich und 11 (27 %) klinisch auffällig. Bei den 6–10-jährigen Patienten sind 2 (18 %) im klinischen Grenzbereich und 9 (82 %) klinisch auffällig. Bei den 0–5-jährigen Geschwisterkindern sind alle 7 (100 %) unauffällig. Bei den 6–10-jährigen Geschwisterkindern ist eines (50 %) im klinischen Grenzbereich und das andere (50 %) klinisch auffällig.

Bei der Gesamtskala des CBCL haben die 6–10-jährigen Patienten höhere T‑Werte (*M* = 76,9, *SD* = 7,1) als die 0–5-jährigen Patienten (*M* = 63,1, *SD* = 12,4), *t* (50) = −3,52, *p* < 0,001.

### Belastungen der Eltern

**Figure 4:**
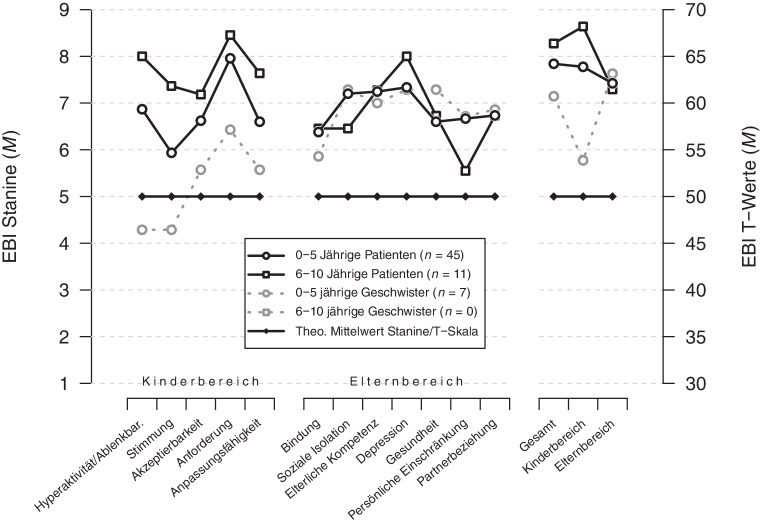

Abb. 4 zeigt die Ausprägung der Elternbelastung bezüglich der einzelnen EBI-Subskalen sowie der Hauptskalen (Gesamt, Kinderbereich, Elternbereich) für die zwei Altersgruppen (0–5-Jährige und 6–10-Jährige) zu Behandlungsbeginn. Um das Belastungsausmaß differenziert beschreiben zu können, wurde zusätzlich zwischen den primären Patienten und den Geschwisterkindern unterschieden.

Die elterliche Gesamtbelastung aus kind- und elternbezogenen Belastungsquellen unterscheidet sich zwischen den Eltern der 0–5-jährigen Patienten und den Eltern der 6–10-jährigen Patienten nicht, *t* (54) = −0,75, *p* = 0,459. Dies gilt auch für den kindsbezogen Belastungsbereich, *t* (54) = −1,75, *p* = 0,087 und den elternbezogenen Belastungsbereich, *t* (54) = 0,19, *p* = 0,846.

**Figure 5:**
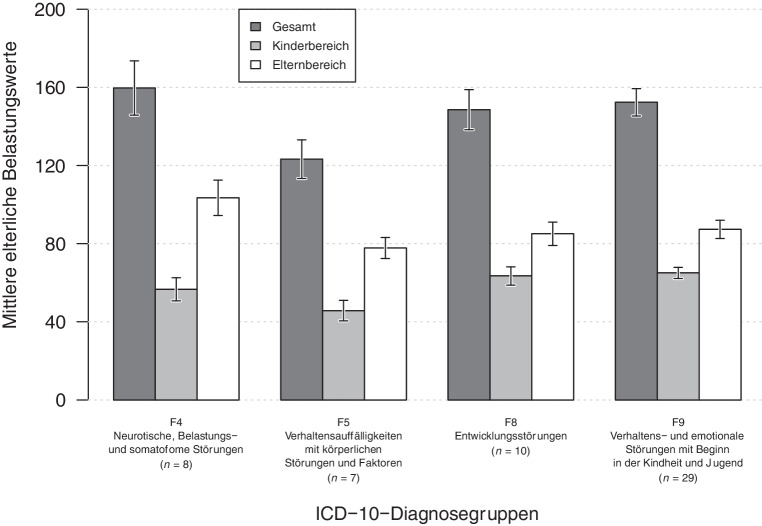

Abb. 5 zeigt die mittleren EBI-Belastungswerte für die entsprechenden Diagnosegruppen des ICD-10 (F4, F5, F8, F9).

Darüber hinaus unterscheidet sich die elterliche Gesamtbelastung hinsichtlich der vier Diagnosegruppen weder in der Gesamtskala des EBI, *F* (4,58) = 1,34, *p* = 0,266, noch im Elternbereich des EBI, *F* (4,58) = 1,44, *p* = 0,232, oder im Kinderbereich des EBI, *F* (4,58) = 2,81, *p* = 0,033.

## Diskussion

Die Ergebnisse der vorliegenden Untersuchung über die Merkmale der bisherigen Inanspruchnahmepopulation der ersten kinderpsychiatrischen Eltern-Kind-Station in Westösterreich verdeutlichen den hohen Bedarf eines interaktionszentrierten Behandlungsangebots für Kinder und ihre Eltern. Im Unterschied zu klassischen kinder- und jugendpsychiatrischen Behandlungsansätzen, die den Fokus primär auf die kindliche Störung ausgerichtet haben, liegt der Schwerpunkt in der Eltern-Kind Behandlung auf der Eltern-Kind-Beziehung. Ziel ist dabei, die Qualität der Eltern-Kind-Interaktion zu verbessern und folglich eine Verminderung der Verhaltensauffälligkeiten der Kinder sowie eine Reduktion des Belastungsausmaßes der Eltern im Umgang mit den Kindern zu bewirken [15]. Zu beachten sind dabei die kinds-, eltern- und interaktionsbezogen Merkmale.

So zeigte sich in unserer Untersuchung hinsichtlich der kindsbezogenen Merkmale, dass die älteren Kinder ab 6 Jahren bei der kindsseitigen Symptomatik des EBI und beim CBCL mehr Symptome aufwiesen als die 0–5-jährigen. Dadurch wird die Bedeutung einer frühzeitigen Erkennung von Verhaltensauffälligkeiten bzw. psychischen Störungen bei sehr jungen Kindern zur Vorbeugung von Chronifizierungen deutlich [25]. Die KiGGS-Studie, welche als wichtigste Studie zur Feststellung von gesundheitsbezogenen Störungen im Kindesalter im deutschsprachigen Raum diskutiert wird [13], verweist ebenfalls auf erhöhte Prävalenzen für psychische Erkrankungen bei den älteren Kindern [12]. Die häufigsten Diagnosen bei Entlassung waren dabei die F98 Verhaltens- oder Emotionale Störungen (19 %), F83 tiefgreifende Entwicklungsstörungen (15 %) und F43 Anpassungsstörungen (15 %). Auch diese Verteilung stimmt mit den Ergebnissen einer bundesweiten Analyse vertragsärztlicher Abrechnungsdaten von 2009–2017 zur Diagnoseprävalenz psychischer Störungen bei Kindern und Jugendlichen in Deutschland [7, 26] überein, bei der Entwicklungsstörungen (F8) die häufigste Diagnose in der Gruppe der Kinder und Jugendlichen (45 % aller psychischen Diagnosen im Jahr 2009) darstellte, gefolgt von Verhaltens- und emotionalen Störungen (F9, ohne F99) und neurotischen, Belastungs- und somatoformen Störungen (F4; 34 % bzw. 10 % aller psychischen Diagnosen). Besonderheiten finden sich auch in der Verwendung von entsprechenden Diagnosemanualen. Die Diagnostik psychischer Störungen der frühen Kindheit soll gemäß der Schlüsselempfehlungen der AWMF-Leitlinien [27] im klinischen Kontext nach der ersten Achse des multiaxialen Klassifikationssystems der ICD-10 (und alle Achsen des MAS der ICD) sowie nach der ersten Achse des DC:0–5 erfolgen [9, 28]. Die Notwendigkeit beider Klassifikationssysteme zeigt sich auch in unserer Stichprobe, da sich manche Störungen sowohl nach ICD-10 und DC:0–5, andere nur nach der ICD-10 (z. B. Enkopresis) und andere nur nach der DC:0–5 (z. B. Beziehungsstörung) klassifiziert werden können.

Betreffend der elterlichen Merkmale zeigte sich, dass die allgemeine Elternbelastung auffällig hoch ist und sich hinsichtlich der verschiedenen Diagnosegruppen nicht unterscheidet. Dies steht im Gegensatz zu den Ergebnissen von Johnston et al., die bei Eltern von Kindern mit Entwicklungsstörungen, ein erhöhtes Maß an elterlichem Stress [29] fanden, welcher im Verlauf persistiert [30]. Da sich die erhobene Elternbelastung in der vorliegenden Untersuchung jedoch hinsichtlich der verschiedenen Diagnosegruppen nicht unterschied, kann davon ausgegangen werden, dass die elterliche Belastung vermutlich eher aus Beziehung und Interaktion entsteht und nicht primär auf eine diagnosespezifische Symptomatik zurückzuführen ist.

Als Fazit ergibt sich daraus, dass eine gemeinsame Behandlung von psychiatrisch auffälligen Kindern mit deren Eltern ein besonderes Behandlungskonzept in der Kinder- und Jugendpsychiatrie darstellt. Wirksamkeitsstudien zu kinderpsychiatrischen Eltern-Kind-Behandlung zeigen auf, dass in der Eltern-Kind-Behandlung signifikante Veränderungen im elterlichen Stresserleben sowie eine Reduktion der kindlichen Symptomatik erzielt wird [11, 31]. Die hohe Elternbelastung in unserer Stichprobe verdeutlicht darüber hinaus den Bedarf von Interventionen auf Elternebene (Einzeltherapie, Paargespräche) sowie die Relevanz einer engen Kooperation zwischen den erwachsenen- und kinderpsychiatrischen Einrichtungen. Es ist davon auszugehen, dass eigene biografische und emotionale Erfahrungen der Eltern einen direkten Einfluss auf ihre Feinfühligkeit im Umgang mit ihren Kindern haben und dadurch die Gefahr einer transgenerationalen Weitergabe von dysfunktionalen Beziehungsmustern besteht (Vgl. Konzept der Gespenster im Kinderzimmer von Freiberg [32]). In vielen Fällen ist aus diesen Gründen eine Zuweisung an eine erwachsenpsychiatrische bzw. psychotherapeutische Behandlung notwendig. Zudem zeigt der hohe Anteil an Geschwisterkinder auf, dass sowohl hinsichtlich der Planung (z. B. Versorgung von Geschwisterkindern, Berufstätigkeit) und der Wahl der Behandlungsform (z. B.: Ausmaß der Interaktionsstörung und Mitbehandlung der Geschwister) eine gemeinsame Absprache zwischen Familie und Institution notwendig ist [15].

Die Ergebnisse zu den Merkmalen der Inanspruchnahmepopulation der kinderpsychiatrischen Eltern-Kind-Station in Tirol verdeutlichen die Chancen und die Notwendigkeit einer Eltern-Kind-Behandlung in der frühen Kindheit, die durch den präventiven Ansatz auch in weiteren Bundesländern mit den entsprechenden strukturellen und personellen Ressourcen implementiert werden sollte.

### Limitationen

Die Limitationen unserer Untersuchung bestehen in der kleinen Stichprobe und der fehlenden Wirksamkeitsüberprüfung mit einem randomisiert-kontrollierten Studiendesign. Andere wesentliche Einflüsse auf die Eltern-Kind-Interaktion und die Belastung der Eltern, wie die Psychopathologie und der Sozialstatus der Eltern oder eine Betreuung durch die Jugendwohlfahrt, wurden nicht miterfasst. Dies soll Gegenstand zukünftiger Forschungsprojekte werden, ebenso wie eine Erfassung der Elternbelastung sowie der kindlichen Symptomatik im Längsschnitt. Darüber hinaus kann aufgrund der kleinen Stichprobengröße keine Differenzierung zwischen Eltern mit einem oder mehreren Kindern vorgenommen werden. Bei der Beurteilung der Ergebnisse zur Prävalenz muss zusätzlich berücksichtigt werden, dass Selektionseffekte auftreten können und eine geringe Vergleichbarkeit der Ergebnisse aufgrund der Methodik (Rekrutierung der Probanden, Erhebungsinstrumente und Diagnosekriterien) möglich ist.
